# Supplementary material for: Transcriptomic and Proteomic Analysis of Marine Nematode Litoditis marina Acclimated to Different Salinities
Source: Genes (Basel). 2022 Apr 7;13(4):651. doi: 10.3390/genes13040651 (PMC9025465; doi:10.3390/genes13040651)
Supplement: Supplementary file 1 [file genes-13-00651-s001.zip › genes-1630146-supplementary/genes-1630146-Revised-Supplementary Materials.pdf]

genes

MDPI

Supplementary Materials

1 Supplementary Figures

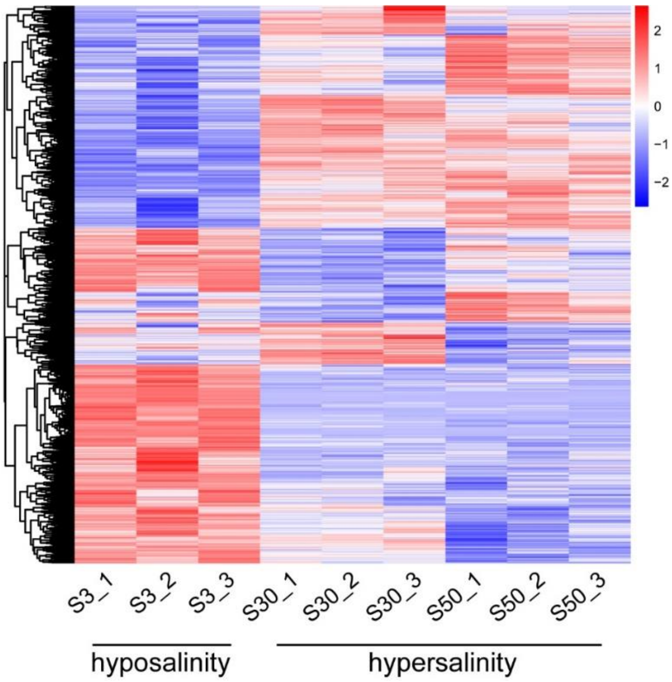

Supplementary Figure S1. Heatmap of expression levels for all DEGs among nine samples. The transition from blue to red strips represents an increase in gene expression levels.

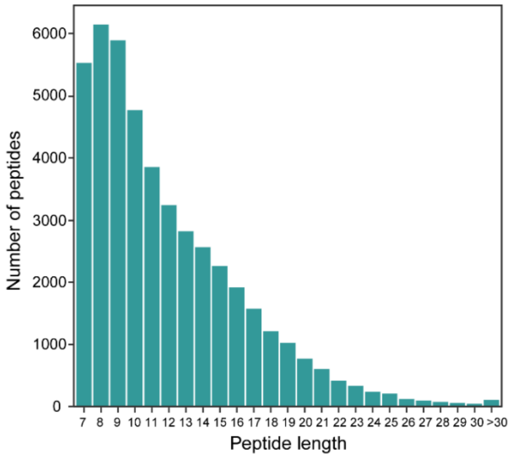

Supplementary Figure S2. Length distribution of peptides identified by mass spectrometry.

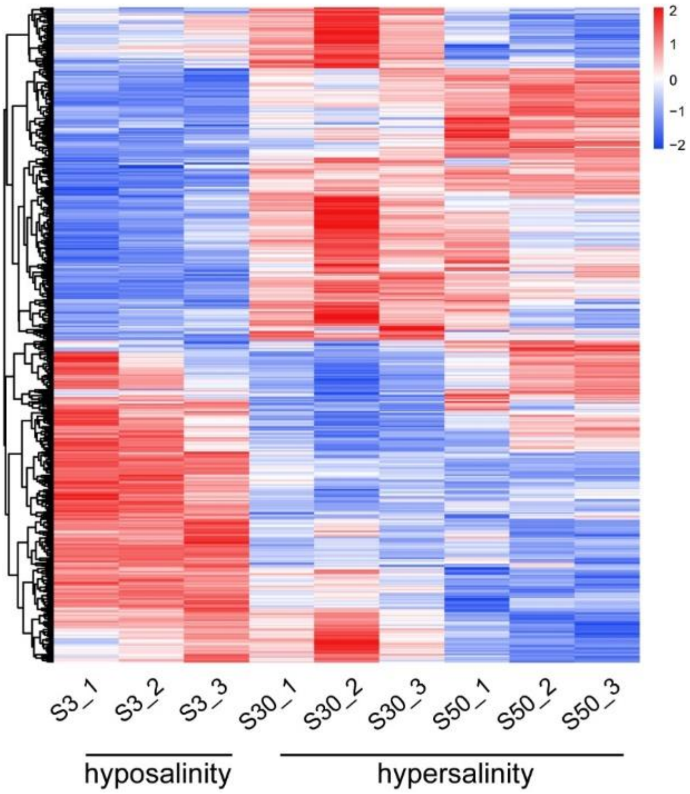

Supplementary Figure S3. Heatmap of expression levels for all DEPs among nine samples. The transition from blue to red strips represents an increase in protein expression levels.

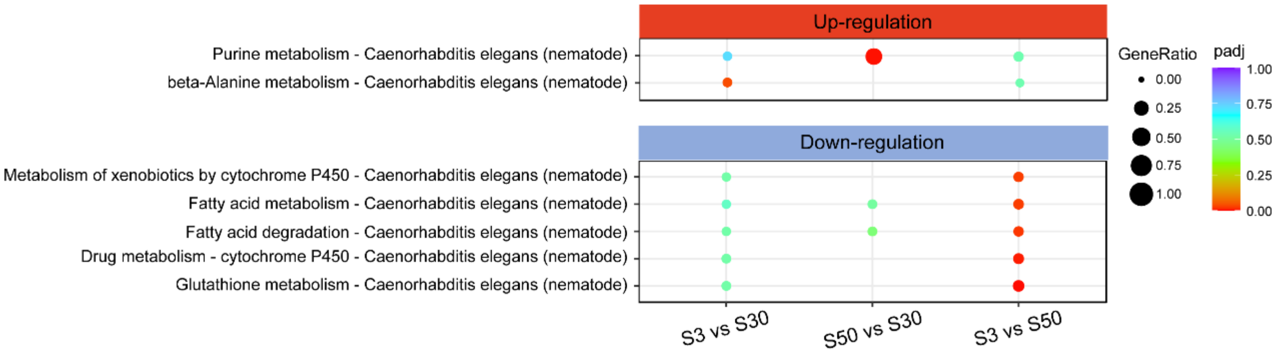

Supplementary Figure S4. KEGG pathway enrichment analysis for DEGs identified via RNA-seq. |log2foldchange| > 1; DESeq2 padj < 0.05 was set as the differential gene screening thresholds. KEGG pathway enrichment analysis of DEGs were achieved by clusterProfiler R package (v3.4.4), an adjusted *P* value (padj) < 0.05 was considered significantly enriched. The color from red to purple represents the significance of the enrichment. GeneRatio was defined as the ratio of the number of differential genes annotated on the KEGG pathway to the total number of differential genes.

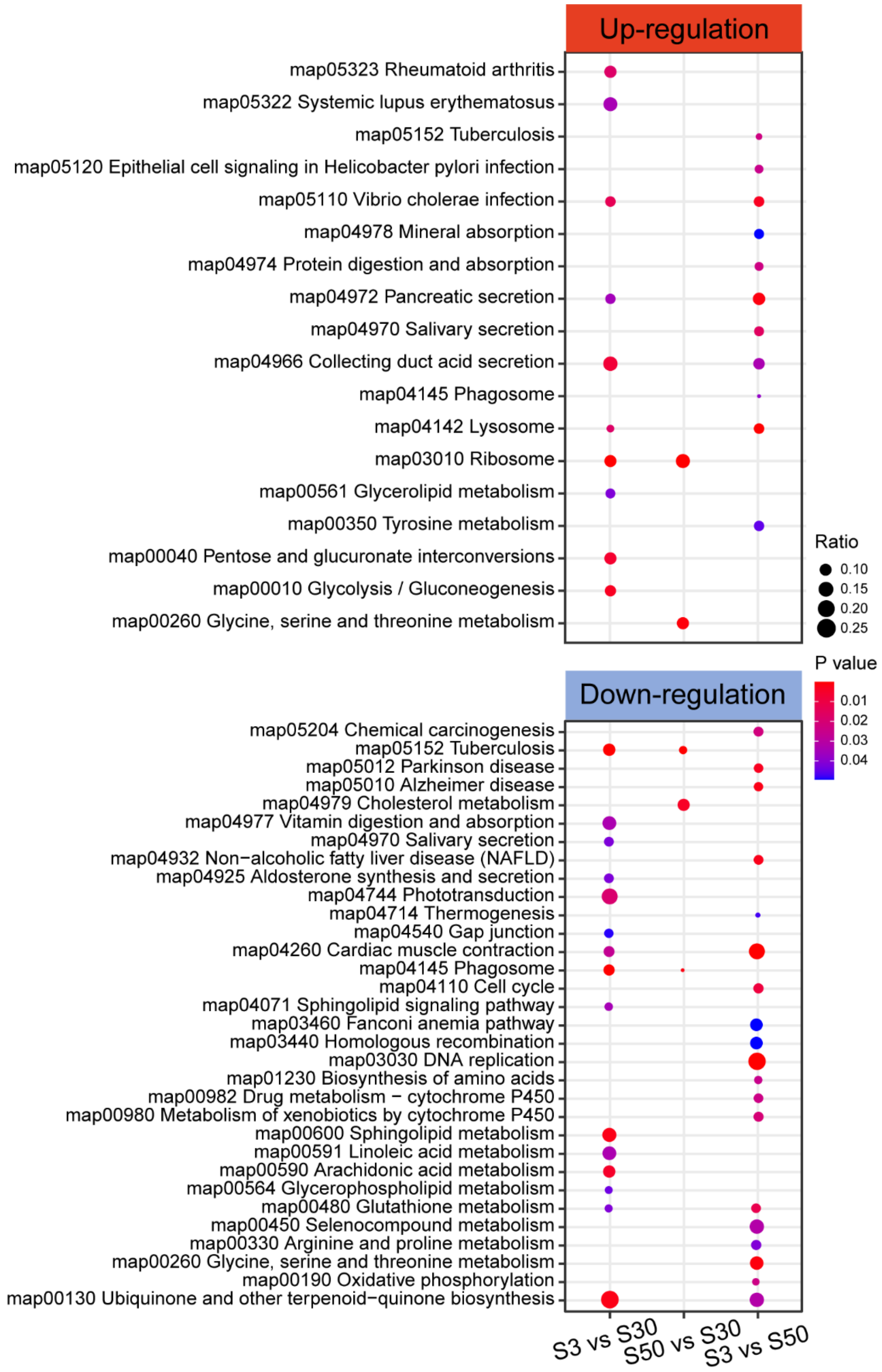

Supplementary Figure S5. KEGG pathway enrichment analysis for DEPs identified via proteomic analysis. Ratio of fold change > 1.3 or < 1/1.3; *P* value < 0.05 was set as the differential protein screening thresholds. KEGG pathway enrichment analysis of DEGs were achieved by Python using an in-house script, a corrected *P* value < 0.05 was considered significantly enriched. The color from red to purple represents the significance of the enrichment. Ratio was defined as the ratio of the number of differential proteins annotated on the KEGG pathway to the total number of differential proteins.

2 Supplementary Tables

Supplementary Table S1. Recipes for agar plates used for different salinity culture conditions.

salinity
low → high

	Media Composition	"S3" NGM	"S30" SW-NGM	"S50" ASW-NGM
Specific	NaCl	3 g		
	seawater		975 ml	
	artificial Sea Salt			50 g
	H_2O	975 ml		975 ml
Common	agar	17 g		
	peptone	2.5 g		
	$CaCl_2$ 1 M	1 ml		
	$MgSO_4$ 1 M	1 ml		
	KH_2PO_4 1 M (pH 6.0)	25 ml		
	cholesterol (5 mg/ml in Ethanol)	1 ml		

Supplementary Table S2. Statistics of mapping ratio.

Sample			Mapping ratio
hyposalinity	S3	1	69.54%
		2	68.57%
		3	70.70%
hypersalinity	S30	1	70.37%
		2	70.05%
		3	70.21%
	S50	1	69.72%
		2	70.36%
		3	69.38%

Clean data were aligned to the *L. marina* reference genome by Hisat2 (v2.0.5, with the default parameters). Mapping ratio for each sample were summarized.

Supplementary Table S3. Overview of the LC-MS/MS analysis results.

Type of data	Number
Total spectrums	306,324
Matched spectrums	78,015
Peptides	45,669
Unique peptides	43,312
Identified proteins	6,068
Quantifiable proteins	5,844

Supplementary Table S4. Expression profiles of enriched tubulin related proteins based on proteomic data via GO enrichment analysis. Red color indicates the expression significance, according to screening thresholds for proteomic or

transcriptomic analysis respectively. Blue color indicates enriched genes that down-regulated in indicated comparison group, while yellow color indicates enriched genes that up-regulated in indicated comparison group.

gene_id	Proteomic level						Transcriptomic level						Gene name
	S3 vs S30		S50 vs S30		S3 vs S50		S3 vs S30		S50 vs S30		S3 vs S50		
	Ratio	P value	Ratio	P value	Ratio	P value	Fold Change	padj	Fold Change	padj	Fold Change	padj	
EVM0000074	0.71	6.68E-05	0.86	0.01	0.82	2.34E-03	0.44	1.84E-07	0.95	0.84	0.46	2.89E-06	*tba-4*
EVM0002197	0.75	2.31E-03	0.90	0.14	0.83	7.04E-03	0.62	0.09	0.74	0.30	0.84	0.64	*tba-4*
EVM0004244	0.73	1.39E-03	1.08	0.14	0.68	4.52E-04	0.67	6.81E-04	0.66	1.63E-03	1.01	0.96	*ben-1*
EVM0006479	0.77	5.58E-03	0.92	0.21	0.83	0.01	0.60	0.07	0.77	0.42	0.78	0.53	*skr-1*
EVM0003257	0.62	8.79E-03	0.65	7.79E-03	0.94	0.43	1.61	3.52E-02	1.29	0.28	1.25	0.22	*lfi-1*
EVM0002519	1.31	0.04	0.58	1.65E-03	2.24	7.07E-04	170.92	1.25E-44	1.30	1.00	131.09	1.34E-50	*lfi-1*
EVM0014264	1.23	1.80E-03	0.93	0.01	1.33	2.29E-04	1.59	1.54E-03	0.95	0.86	1.67	7.27E-04	*ben-1*
EVM0013178	0.96	0.53	1.38	0.02	0.70	0.02	0.34	1.78E-09	0.73	0.10	0.47	2.20E-04	*unc-59*
EVM0002084	1.56	2.06E-03	1.38	0.01	1.13	0.08	0.96	0.82	0.88	0.60	1.08	0.74	*dnc-1*

GO:0005819, spindle (EVM0000074, EVM0002197, EVM0004244, EVM0003257); GO:0005874, microtubule (EVM0000074, EVM0002197, EVM0004244, EVM0003257, EVM0002519); GO:0005876, spindle microtubule (EVM0003257, EVM0002519, EVM0014264); GO:0015630, microtubule cytoskeleton (EVM0000074, EVM0002197, EVM0004244, EVM0006479, EVM0003257); GO:0030496, midbody (EVM0013178, EVM0002084); GO:0072687, meiotic spindle (EVM0000074, EVM0002197).

Supplementary Table S5. 29 out of 78 low-salinity-specific genes showed specific induction upon hyposaline stress in

L. marina based on our previous data [39].

Transcriptomic level

gene_id	0.3% vs 3%		0.3% vs 6%		6% vs 3%		Gene name
	Fold Change	padj	Fold Change	padj	Fold Change	padj	
EVM0017308	147.29	3.92E-10	32.86	3.07E-17	4.49	1.00	*T02B11.3*
EVM0008374	44.85	6.38E-82	25.62	1.67E-96	1.77	0.24	*mca-1*
EVM0000094	31.37	8.65E-156	26.56	1.11E-216	1.18	0.59	*twk-33*
EVM0008743	6.99	3.16E-10	5.00	1.02E-08	1.38	0.62	*R04B5.5*
EVM0010567	5.92	2.69E-19	4.18	9.73E-87	1.41	0.21	*F52E1.14*
EVM0007934	3.80	2.56E-33	3.11	1.22E-30	1.22	0.30	*vha-5*
EVM0004638	3.79	2.03E-06	2.01	1.46E-04	1.87	0.06	*ttr-30*
EVM0001362	3.06	1.99E-21	5.52	2.21E-84	0.55	1.23E-04	--
EVM0011529	3.01	2.73E-27	3.04	1.38E-42	0.99	0.96	*sfxn-1.2*
EVM0015459	2.96	7.32E-13	2.12	8.28E-40	1.39	0.07	*tatn-1*
EVM0011489	2.63	8.59E-22	2.60	1.61E-47	1.01	0.98	*K07C11.8*
EVM0013037	2.60	1.14E-11	2.73	2.49E-64	0.95	0.83	*T16G1.6*
EVM0016452	2.58	1.54E-09	1.95	6.49E-17	1.32	0.17	*tps-2*
EVM0017439	2.44	5.10E-11	2.65	5.20E-39	0.92	0.70	*pnp-1*
EVM0014277	2.39	2.32E-03	3.32	1.34E-07	0.72	0.47	*F36G3.2*
EVM0006956	2.20	5.68E-04	2.94	8.46E-17	0.75	0.36	*F36G3.2*
EVM0015999	2.08	1.68E-05	2.02	4.29E-11	1.03	0.94	*dmd-7*
EVM0002515	2.06	6.65E-05	1.80	1.66E-04	1.14	0.65	*R02C2.7*
EVM0002057	2.02	2.54E-04	2.68	9.85E-17	0.75	0.27	*Y57A10A.26*
EVM0003972	2.01	1.64E-04	1.81	1.38E-03	1.11	0.77	*ttr-15*
EVM0011338	1.92	3.84E-04	2.60	1.07E-25	0.74	0.19	--
EVM0001780	1.87	3.09E-15	1.76	8.37E-28	1.06	0.68	*gdh-1*
EVM0006214	1.78	0.02	1.70	0.03	1.05	0.92	*Y71F9B.9*
EVM0007619	1.62	7.18E-04	1.55	5.36E-04	1.04	0.88	*Y43F8C.13*
EVM0016161	1.58	2.75E-04	2.28	1.06E-18	0.69	0.02	*C01B10.3*
EVM0004057	1.51	7.08E-06	1.87	3.71E-06	0.81	0.17	*F08F3.4*
EVM0012344	1.51	0.01	2.51	1.22E-29	0.60	3.77E-03	*bus-2*
EVM0003311	1.44	0.01	1.23	0.01	1.16	0.39	*C23H3.2*
EVM0009105	1.40	6.21E-03	1.42	3.48E-06	0.98	0.92	*C44E4.5*

0.3%: 0.3% salinity, hyposaline stress condition;
3%: 3% seawater salinity, control;
6%: 6% salinity, hypersaline stress condition.

Supplementary Table S6. 11 out of 69 high-salinity-specific genes showed specific induction upon hypersaline stress in *L. marina* based on our previous data [39].

gene_id	Transcriptomic level: 6% vs 3% Fold Change	Transcriptomic level: 6% vs 3% padj	Transcriptomic level: 6% vs 0.3% Fold Change	Transcriptomic level: 6% vs 0.3% padj	Transcriptomic level: 0.3% vs 3% Fold Change	Transcriptomic level: 0.3% vs 3% padj	Gene name
EVM0012459	2.95	4.77E-11	2.16	6.01E-14	1.37	0.10	*Y12A6A.1*
EVM0011921	2.74	5.24E-20	3.44	2.12E-62	0.80	0.04	*F47G3.4*
EVM0012075	2.35	2.41E-09	2.26	1.65E-20	1.05	0.84	*T10B10.4*
EVM0009309	2.08	1.67E-05	1.80	7.38E-12	1.16	0.50	*clec-62*
EVM0013159	2.08	6.60E-04	2.22	2.11E-10	0.94	0.85	*F37C4.6*
EVM0001663	2.06	5.12E-06	2.05	2.88E-11	1.01	0.98	*gpdh-1*
EVM0008192	1.85	1.30E-03	1.62	1.56E-06	1.15	0.57	*clec-48*
EVM0013254	1.74	4.46E-08	2.21	1.88E-36	0.79	0.03	*col-160*
EVM0011220	1.64	1.44E-06	2.36	1.42E-42	0.70	8.91E-04	*Y71G12B.10*
EVM0000243	1.38	4.78E-02	3.50	1.35E-37	0.39	1.42E-10	*snf-9*
EVM0008918	1.36	1.82E-04	2.08	1.72E-57	0.66	1.54E-08	*lrp-1*

0.3%: 0.3% salinity, hyposaline stress condition;
3%: 3% seawater salinity, control;
6%: 6% salinity, hypersaline stress condition.

Supplementary Table S7. Expression profiles of 47 annotated transthyretin-like (TTL) family genes in *L. marina*.

gene_id	Transcriptomic level				Proteomic level				Gene name
	S3 vs S30		S3 vs S50		S3 vs S30		S3 vs S50		
	Fold Change	padj	Fold Change	padj	Ratio	*P* value	Ratio	*P* value	
* EVM0004800	0.64	2.83E-04	0.54	9.22E-05	0.74	6.01E-02	0.93	5.81E-01	*ttr-16*
* EVM0003282	1.03	9.13E-01	0.95	8.73E-01	0.80	4.59E-02	1.13	2.73E-01	*ttr-41*
* EVM0003534	0.83	4.96E-01	0.56	1.05E-02	NA	NA	NA	NA	*ttr-15*
* EVM0003838	1.13	5.88E-01	1.44	5.90E-02	0.97	7.94E-01	1.40	1.56E-02	*ttr-46*
EVM0003584	0.89	5.02E-01	0.84	4.10E-01	0.92	3.95E-01	1.21	1.36E-01	*ttr-48*
EVM0004593	1.54	1.83E-03	1.11	5.67E-01	0.80	9.65E-03	0.97	3.34E-01	*ttr-27*
EVM0002004	0.61	4.45E-03	0.46	3.68E-05	0.66	1.77E-02	0.85	1.52E-01	*ttr-51*
EVM0001487	1.10	4.36E-01	0.97	8.74E-01	0.65	1.44E-02	0.85	1.46E-02	*ttr-27*
EVM0008675	0.97	8.51E-01	0.83	2.52E-01	0.76	3.89E-02	0.97	6.79E-01	*ttr-5*
* EVM0016920	0.85	3.70E-01	0.36	9.72E-08	0.87	1.93E-01	1.05	2.37E-01	*ttr-18*
EVM0008582	0.98	9.49E-01	0.80	1.82E-01	0.89	6.01E-02	0.99	7.70E-01	*ttr-47*
EVM0004484	1.14	5.53E-01	0.96	9.12E-01	0.72	6.26E-02	0.96	5.40E-01	*ttr-35*
EVM0012475	0.69	1.61E-02	0.39	1.02E-09	0.83	1.62E-01	1.08	4.01E-01	*ttr-31*
* EVM0016470	1.41	2.81E-02	1.46	5.06E-02	1.09	4.28E-01	1.52	1.98E-03	*ttr-59*
* EVM0015122	0.77	3.00E-01	0.72	1.38E-01	0.76	1.72E-02	0.88	2.41E-01	*ttr-36*
* EVM0005705	1.25	2.85E-01	0.62	7.47E-03	0.97	7.75E-01	1.38	1.43E-02	*ttr-5*
* EVM0017357	0.55	1.58E-03	0.58	8.89E-03	0.90	4.76E-01	1.61	4.28E-03	*ttr-44*
* EVM0005297	0.37	3.62E-09	0.25	1.73E-13	0.68	2.30E-02	0.80	1.00E-01	*ttr-44*
EVM0014605	0.92	8.10E-01	0.49	9.90E-04	0.97	8.13E-01	1.22	1.54E-01	*ttr-8*
* EVM0010840	10.95	1.74E-37	11.96	4.97E-37	0.80	1.01E-01	1.02	7.48E-01	*ttr-15*
EVM0010105	0.55	4.00E-05	0.52	4.88E-04	NA	NA	NA	NA	*ttr-44*
* EVM0010790	0.89	7.64E-01	0.54	9.53E-03	0.84	1.54E-01	1.07	4.81E-01	*ttr-33*
* EVM0008626	1.45	1.90E-02	1.19	4.31E-01	1.11	1.11E-01	1.48	2.24E-03	*ttr-46*
EVM0005032	0.71	2.63E-01	0.73	3.79E-01	1.01	9.46E-01	1.33	2.50E-02	*ttr-7*
* EVM0004159	1.55	5.94E-02	1.72	2.04E-02	0.98	7.71E-01	1.12	1.92E-01	*ttr-59*
EVM0007550	0.75	4.58E-01	0.56	3.76E-02	0.76	4.18E-02	0.97	6.63E-01	*ttr-30*
EVM0011170	0.54	4.54E-02	0.55	4.45E-02	0.53	1.03E-02	0.60	1.31E-03	*ttr-27*
EVM0009470	2.07	4.00E-04	1.77	5.66E-03	0.83	1.13E-01	1.05	4.92E-01	*ttr-53*
EVM0003549	0.90	7.55E-01	0.66	8.86E-02	NA	NA	NA	NA	*ttr-52*
EVM0010505	1.37	3.09E-01	1.64	8.13E-02	NA	NA	NA	NA	*ttr-44*
EVM0003972	16.96	1.75E-15	14.33	2.22E-15	1.72	2.28E-03	2.60	2.65E-04	*ttr-15*
EVM0004658	0.004	1.43E-08	0.003	1.29E-10	1.00	9.47E-01	1.48	1.29E-03	*ttr-32*
* EVM0004638	6.15	1.64E-08	3.28	5.32E-05	1.52	1.13E-02	1.68	1.49E-03	*ttr-30*
EVM0016217	2.13	4.98E-02	3.32	1.64E-04	NA	NA	NA	NA	*ttr-54*
EVM0004621	1.05	9.49E-01	1.08	9.21E-01	NA	NA	NA	NA	*ttr-38*
* EVM0006931	0.42	2.17E-01	0.26	2.43E-02	NA	NA	NA	NA	*ttr-49*
EVM0008749	0.09	1.59E-02	0.06	1.39E-03	NA	NA	NA	NA	*ttr-40*
EVM0012444	0.72	8.17E-01	0.73	8.44E-01	NA	NA	NA	NA	*ttr-29*
EVM0012210	0.57	6.94E-01	5.28	3.04E-01	NA	NA	NA	NA	*ttr-46*
EVM0003766	0.07	6.15E-02	0.23	5.74E-01	0.98	7.55E-01	0.88	2.56E-01	*ttr-46*
EVM0004005	18.59	1.01E-01	19.03	9.04E-02	NA	NA	NA	NA	*ttr-44*
EVM0013265	5.65	1.00E+00	0.79	9.29E-01	NA	NA	NA	NA	*ttr-14*
EVM0004803	1.95	1.00E+00	3.88	1.00E+00	NA	NA	NA	NA	*ttr-3*
EVM0010813	3.71	1.00E+00	3.80	1.00E+00	NA	NA	NA	NA	*ttr-29*
EVM0016694	NA	NA	NA	NA	NA	NA	NA	NA	*ttr-2*
EVM0012079	NA	NA	NA	NA	NA	NA	NA	NA	*ttr-30*
EVM0000816	NA	NA	NA	NA	NA	NA	NA	NA	*ttr-45*

Red color indicates the significance in expression at either mRNA level or protein level, or both, for the indicated TTL gene. Genes marked with an asterisk were significantly induced in response to both low and high salinity stresses, as reported in our previous study [39].

Supplementary Table S8. Up-regulation of small GTPase-related genes at transcriptional level under low-salinity condition.

gene_id	Transcriptomic level				Proteomic level				Gene name
	S3 vs S30		S3 vs S50		S3 vs S30		S3 vs S50		
	Fold Change	padj	Fold Change	padj	Ratio	*P* value	Ratio	*P* value	
EVM0001914	4.04	8.89E-29	3.75	6.81E-17	1.00	8.81E-01	1.07	2.21E-01	*exc-5*
EVM0002411	115.13	9.20E-26	353.07	3.60E-14	1.04	4.21E-01	1.11	4.63E-02	*efa-6*
EVM0004317	1.61	4.72E-07	2.42	2.78E-11	0.92	2.13E-01	1.03	4.64E-01	*itsn-1*
EVM0004341	2.05	1.67E-07	2.64	1.67E-09	NA	NA	NA	NA	*tag-77*
EVM0008542	1.53	9.60E-04	2.47	2.53E-10	NA	NA	NA	NA	*unc-73*
EVM0010248	2.11	2.19E-06	2.55	9.93E-08	0.94	5.50E-03	0.98	5.37E-01	*Y37A1B.17*
EVM0012791	2.62	1.16E-08	4.07	3.64E-16	NA	NA	NA	NA	*frm-3*
EVM0014485	351.09	1.32E-10	64.23	1.77E-17	NA	NA	NA	NA	*R05G6.10*
EVM0015120	2.13	1.29E-10	3.38	1.44E-12	NA	NA	NA	NA	*tiam-1*

3 Supplementary Files

Supplementary File S1. Identified DEGs via RNA-seq analysis.
Supplementary File S2. Identified DEPs via proteomic analysis.
Supplementary File S3. Sixty-six genes expressed proportionally to environmental salinity.
